# Impact of radiation dose distribution on nutritional supplementation needs in head and neck cancer radiotherapy: a voxel-based machine learning approach

**DOI:** 10.3389/fonc.2024.1346797

**Published:** 2024-02-28

**Authors:** Sudharsan Madhavan, Mauricio Gamez, Yolanda I. Garces, Scott C. Lester, Daniel J. Ma, Daniel W. Mundy, Michelle A. Neben Wittich, Jing Qian, David M. Routman, Robert L. Foote, Satomi Shiraishi

**Affiliations:** Department of Radiation Oncology, Mayo Clinic, Rochester, MN, United States

**Keywords:** voxel-based analysis, head and neck cancer, outcomes modeling, feeding tube, explainable machine learning, larynx, pharyngeal constrictor muscles, weight loss

## Abstract

**Objectives:**

To investigate the relationship between nutritional supplementation and radiation dose to the pharyngeal constrictor muscles and larynx for head and neck (HN) cancer patients undergoing radiotherapy.

**Methods:**

We retrospectively analyzed radiotherapy (RT) dose for 231 HN cancer patients, focusing on the pharyngeal constrictors and larynx. We defined nutritional supplementation as feeding tube utilization or >10% weight loss from baseline within 90 days after radiotherapy completion. Using deformable image registration (DIR), we mapped each patient’s anatomical structures to a reference coordinate system, and corresponding deformations were applied to dose matrices. Voxel doses were utilized as features for ridge logistic regression models, optimized through 5-fold cross-validation. Model performance was assessed with area under the curve of a receiver operating curve (AUC) and F1 score. We built and compared models using 1) pharyngeal constrictor voxels, 2) larynx voxels, 3) clinical factors and mean regional dose metrics, and 4) clinical factors and dose-volume histogram metrics. Test set AUCs were compared among the models, and feature importance was evaluated.

**Results:**

DIR of the pharyngeal constrictors and larynx yielded mean Dice coefficients of 0.80 and 0.84, respectively. Pharyngeal constrictors voxels and larynx voxel models had AUC of 0.88 and 0.82, respectively. Voxel-based dose modeling identified the superior to middle regions of the pharyngeal constrictors and the superior region of larynx as most predictive of feeding tube use/weight loss. Univariate analysis found treatment setting, treatment laterality, chemotherapy, baseline dysphagia, weight, and socioeconomic status predictive of outcome. An aggregated model using mean doses of pharyngeal constrictors and larynx subregions had an AUC of 0.87 and the model using conventional DVH metrics had an AUC of 0.85 with p-value of 0.04. Feature importance calculations from the regional dose model indicated that mean doses to the superior-middle pharyngeal constrictor muscles followed by mean dose to the superior larynx were most predictive of nutritional supplementation.

**Conclusions:**

Machine learning modeling of voxel-level doses enables identification of subregions within organs that correlate with toxicity. For HN radiotherapy, doses to the superior-middle pharyngeal constrictors are most predictive of feeding tube use/weight loss followed by the doses to superior portion of the larynx.

## 1 Introduction

Swallowing difficulties are a prevalent side effect of radiotherapy (RT) treatments for head and neck cancers (1–5). RT involves targeting cancers with a three-dimensional (3D) radiation dose. This often leads to the incidental irradiation of nearby organs that play a role in swallowing. In contemporary practices, radiotherapy treatment plans condense the 3D dose distributions inside delineated organs into two-dimensional dose-volume histograms (DVHs). Specific metrics for organs at risk (OARs) and target volumes are scrutinized to reduce the chance of adverse side effects. Physicians qualitatively assess the spatial distribution of radiation doses, focusing on regions that may pose potential toxicities or affect target coverage. This method has proven effective, with a number of toxicities linked to DVH metrics through various normal tissue complication probability (NTCP) modeling, as evidenced in QUANTEC and various clinical trials (1, 5–10). For example, Mavroidis et al. (11), showed that generalized mean dose for superior pharyngeal constrictors to be most predictive of dysphagia at 6 months post-RT. Using Lyman Kutcher Burman (LKB) model which is a popular methodology accounting for seriality of the OAR (12, 13), their study reported D_50_ (dose at which there is 50% chance of complication) of 62.0 Gy, slope parameter m = 0.1, and dose-volume parameter n = 0.49 with AUC of 0.74 for superior pharyngeal constrictor muscles, suggesting moderate sensitivity to subregion damage (14). However, when relying exclusively on DVHs for analysis, delineation of the OARs is necessary, and it is presupposed that every section of an organ has an equal sensitivity to radiation and contributes identically to the overall risk of toxicity. Additionally, Samant et al. reported that machine learning (ML) models can often quantify NTCP better than LKB models, motivating explorations of ML approaches for toxicity analysis (15).

Furthermore, recent studies have shown that the toxicity observed can depend on which subregion of a segmented organ was irradiated (2–4, 16–37). Functionally distinct subregions within a single OAR contour may not be accounted for in current treatment planning. For example, Jiang et al. used ML to identify subregions within the parotid and submandibular glands that correlated with xerostomia (22). Another study by Eisbruch et al. found the pharyngeal constrictor muscles and glottic/supraglottic larynx subsites were most dysphagia-related using videofluoroscopy (38). In clinical practice, it is common for glottic, supraglottic, and subglottic larynx subregions to be grouped under a single larynx segmentation. Likewise, the superior, middle, and inferior pharyngeal constrictor muscles are collectively evaluated as one entity during treatment planning. In addition to the potential presence of distinct subregions, there is a growing number of studies on voxel-based optimization of radiation treatment plans (39–41). These studies have used voxel-based objectives for optimization and opens an opportunity to reflect spatial dose constraints during treatment planning.

Conventionally, investigation of sub-regions of an OAR required radiomics texture extraction based on images and/or time-consuming and resource-intensive manual contouring of each region (42). Manual contouring is also subject to inter-observer variations and inconsistent implementation of contouring guidelines (43–45). In this study, we employed a machine-learning approach to determine the radiation dose’s voxel-wise correlation with malnutrition, pinpointing distinctive features in the pharyngeal constrictors and larynx sub-regions without preliminary contouring. We focused on interpretable feature importance to analyze the spatial dose dependency within an organ. This may eventually inform dose sparing of a subregion during treatment planning to reduce malnutrition risk. While methodologies akin to ours have been used in head and neck RT studies focusing on toxicities such as xerostomia and acute dysphagia (28, 29), to the best of our knowledge, there has not been an investigation into the 3D dose distribution’s impact on malnutrition, measured by feeding tube (FT) utilization and weight loss. Furthermore, predicting the need for a feeding tube early in radiotherapy can also enhance patient care. Although prophylactic FT placement is occasionally advised (46), its blanket application can be unnecessary or harmful. About half of the preemptively inserted FTs prove marginally beneficial (47), with complications such as infections being prevalent (48). Moreover, FT placement postpones the transition back to regular diets (49), adversely affecting long-term well-being (18, 50, 51). Therefore, identifying patients in true need of FT can improve quality of care.

## 2 Materials and methods

### 2.1 Cohort selection

After Institutional Review Board approval, we conducted a retrospective analysis of 352 patients treated for head and neck cancer at our institution from January 2016 to November 2020, all of whom had granted consent for their medical records to be used in research. We accessed our department’s patient outcomes database (52) and filtered for patients based on International Classification of Diseases (ICD)-9 and 10 codes (53, 54) specific to cancers in the salivary glands, oropharynx, oral cavity, nasopharynx, nasal cavity, sinuses, larynx, and hypopharynx. We excluded patients based on the following criteria: absence of baseline dysphagia assessment before radiotherapy (60 patients), FT insertion before RT (34 patients), radiation doses outside the range of 1.2-2.2 Gy/fraction, discernible disfigurement of the pharyngeal constrictors and larynx due to disease or surgery, and prior RT in an area with potential overlap (27 patients). Ultimately, 231 patients, treated with either photon or proton radiotherapy with prescription dose in the range of 30-81.6 Gy delivered in 15-68 fractions, were deemed suitable for this study.

### 2.2 Data collection

**Table 1** summarizes the clinical variables studied in our analysis. We sourced data from our institution’s electronic health record reporting database, focusing on parameters such as gender, feeding tube usage, weight, birth date, and the primary address’s 9-digit zip code. Feeding tube utilization data was gathered by looking for procedure codes corresponding to the insertion of stomach, gastrostomy, or jejunostomy tubes. Patients who had a feeding tube before starting RT were excluded based on the procedure date, as such utilization is likely attributable to surgery, disease, or both as opposed to RT. Socioeconomic status was inferred using the Area Deprivation Index by Kind and Buckingham (55), derived from the zip code associated with the patient’s primary address. This index, ranging from 1 to 100, gauges the socioeconomic disadvantage of a neighborhood, with higher scores denoting greater disadvantage. We also collected baseline dysphagia grades assessed by the care team following the Common Terminology Criteria for Adverse Events (CTCAE v4.03) within ±2 weeks of radiotherapy initiation (56, 57). Additionally, data on smoking habits, concurrent chemotherapy, and treatment context (either primary or post-operative RT) was obtained via chart reviews. **Table 1** provides information on clinical variables and on treatment sites of the patients included in the study.

**Table 1:** List of clinical parameters investigated, and treatment site of the patient cohort considered in this study.

Clinical variables		Value
Baseline dysphagia grade	Grades 0-1	208
	Grades 2-3	23
Age at RT start	Mean	60.85 years
	Range	[23, 89] years
ADI	Mean	45.01
	Range	[1, 98]
Treatment setting	Surgery before RT	146
	No surgery before RT	85
Smoker status	Smoker	120
	Never smoker	111
Gender	Male	189
	Female	42
Radiation type	Photon	131
	Proton	100
Concurrent chemotherapy	Yes	156
	No	75
Treatment site (% of FT/WL)	Salivary glands	6 (50%)
	Oropharynx	155 (54%)
	Oral cavity	23 (61%)
	Nasopharynx	12 (75%)
	Nasal cavity and sinuses	15 (33%)
	Larynx	17 (35%)
	Hypopharynx	3 (67%)

All radiotherapy treatments were planned in the Eclipse treatment planning system (Siemens Healthineers company, Erlangen, Germany). Physical radiation doses in proton plans were scaled by 1.1 (58–60) to account for relative biological effect compared to photon plans. Photon treatments were generally planned to use two to four volumetric modulated arcs. Proton treatments were planned with a pencil beam scanning method utilizing two to five static fields. From our planning system, we exported DICOM files corresponding to radiation doses, CT scans, and structural sets. Of note, the head and neck anatomy of these data sets were retrospectively segmented consistently by a specially trained team of physicians and medical dosimetry assistant as part of a separate project (61). The contouring followed the consensus guidelines in Brouwer et al. (62).

### 2.3 Endpoint definition

The endpoint used to characterize malnutrition was feeding tube (FT) utilization or >10% weight loss from baseline within 90 days after radiotherapy completion. Though FT usage typically arises when a patient sheds over 10% of their initial weight, some clinicians and/or patients opt against it on a case-by-case basis. Our practice is to only recommend a FT if it is clinically indicated (typically >10% weight loss from baseline). We do not use FT prophylactically. Regardless, such patients remain malnourished, indicating a toxicity affecting their quality of life. We chose to merge these observations and study them as one endpoint; for the remainder of the paper, this endpoint will be referred to as FT/WL for simplicity. The baseline weight was defined as the weight recorded closest to the RT commencement, ensuring it was within a ±2-week window of that date.

### 2.4 Analysis

#### 2.4.1 Overview

**Figure 1:**
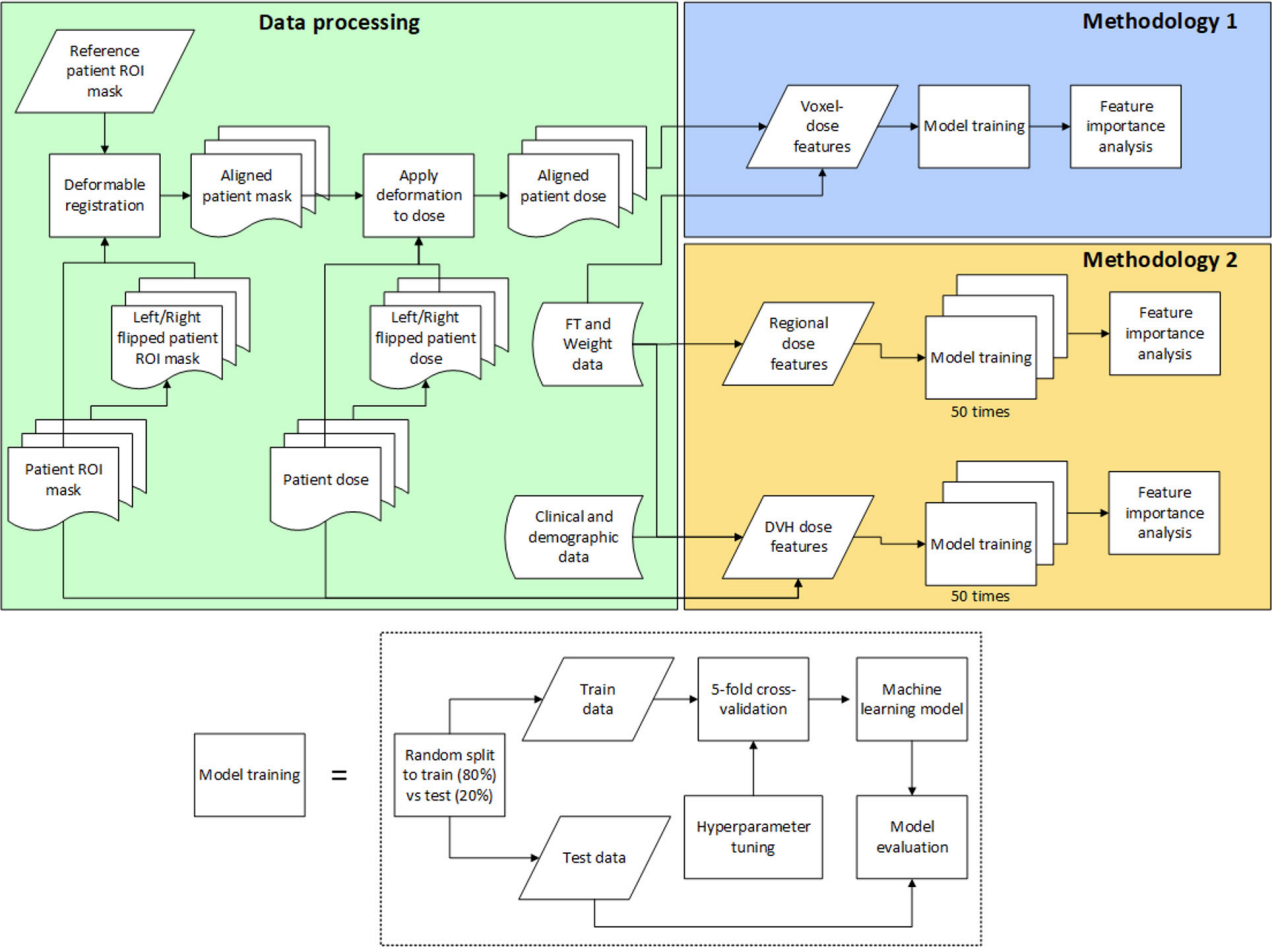
Flow diagram of the analysis.

As illustrated in **Figure 1**, this analysis had two different methodologies after data processing. In the first methodology, 3D voxel-based dose models were trained to identify regions within the pharyngeal constrictors and larynx that better differentiated toxicity endpoints. Because the voxel-based model utilized a large number of input voxels (22,020 for pharyngeal constrictors and 20,814 for larynx), it was prone to overfitting. To validate and confirm the subregion findings, the second methodology investigated models with reduced features where mean doses from segmented sub-regions of the pharyngeal constrictor muscles and larynx along with clinical variables (14 input features). Similarly, another model that combined DVH metrics used in our clinic with the identical clinical parameters (13 input features) was studied as a comparison.

#### 2.4.2 Data preprocessing

**Figure 2:**
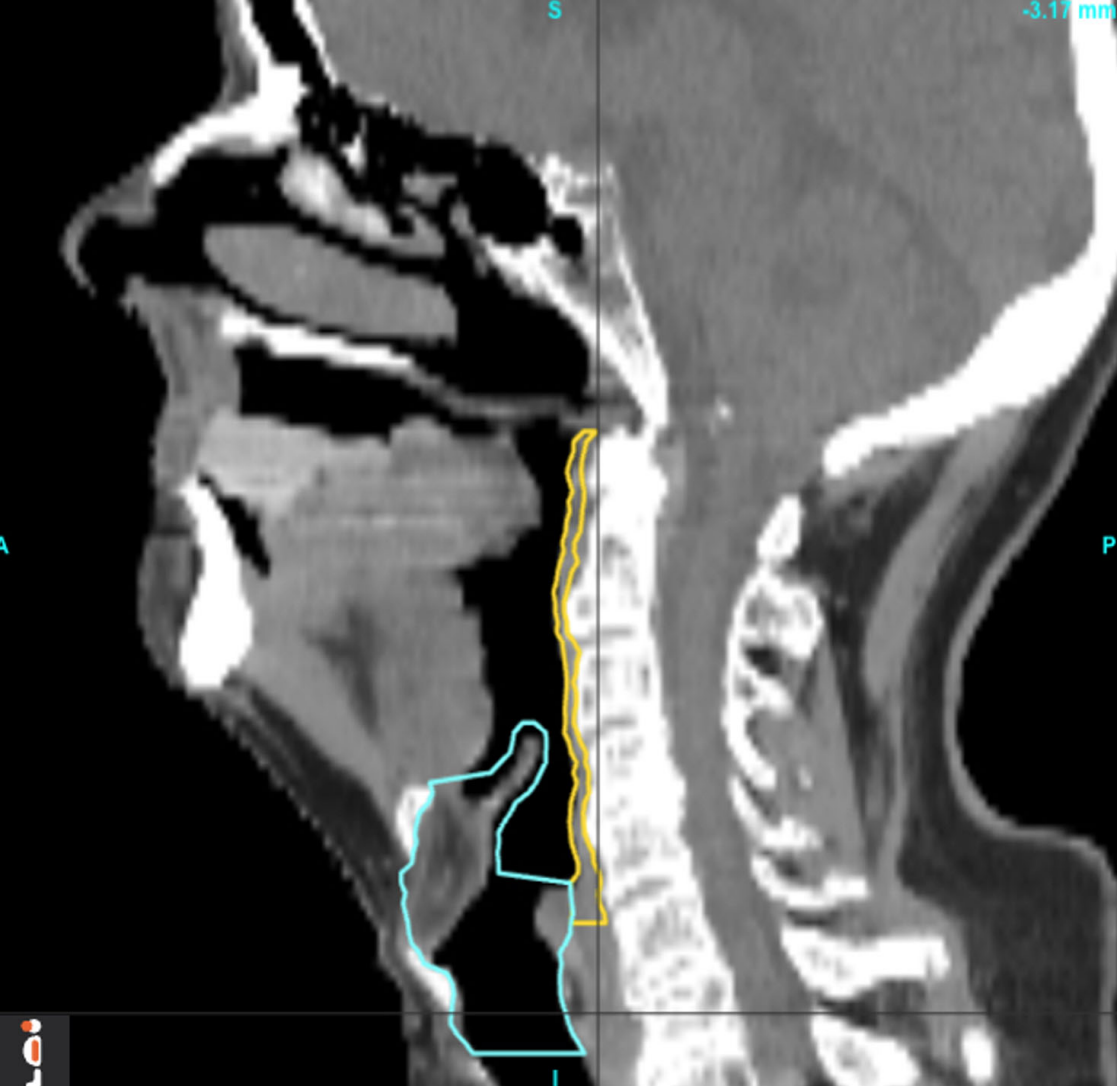
Pharyngeal constrictor muscles and larynx of the reference patient.

All patients’ pharyngeal constrictors and larynx were deformably registered to a reference patient’s corresponding organs to align them in the same coordinate system. The reference patient shown in **Figure 2**, was chosen based on having a larynx structure size close to the population average, as well as the absence of any disfigurement due to prior surgery or disease involving the pharyngeal constrictors and/or larynx. Deformable registration was performed using the open-source package, Elastix (63–65). The planning images of most patients included in the study had a native resolution of 1.27 x 1.27 mm^2^ in the axial direction, with slice thicknesses of either 1 or 2 mm. To maintain uniformity, all images were interpolated to a resolution of 1.27 mm x 1.27 mm x 1 mm in the coronal, sagittal, and axial directions, respectively. The DICOM images (66) were then cropped around organ +4 mm using the open-source packages DicomRTTool and ANTs (67, 68). Rigid, affine, and deformable transformations were subsequently applied. The quality of image registrations was evaluated using the Dice coefficient. For the 3D models, we also augmented the training dataset by flipping the OAR contours and dose left-right to create mirrored dose distributions. This is based on the premise that both the pharyngeal constrictors and larynx are midline structures and there are no laterality preferences for one versus the other. The flipped contour and doses were registered to the reference coordinate system in the same manner as the original data. After the deformable image registrations, the same deformation was applied to each patient’s dose matrix to obtain dose within the reference coordinate system. Dose to each voxel was extracted, and each numerical input feature was standardized. This standardization process involved adjusting each feature such that it had a mean of zero and a standard deviation of one among the training set. Standardization ensures that our model’s performance is not biased by variations in the absolute dose levels, but rather focuses on the relative differences in dose distribution. All categorical features were one-hot encoded and expressed in terms of zeros and ones.

#### 2.4.3 Modeling and statistical analysis

We initially evaluated ridge logistic regression, eXtreme Gradient Boosting (XGBoost), and Light Gradient Boosting Machine (LightGBM) algorithms for modeling. Ridge logistic regression was implemented using the open-source package cuML (69) and scikit-learn (70), while XGBoost (71) and LightGBM (72) models were utilized from their respective repositories. We performed voxel-based analysis using these algorithms with a subset of cohort, and areas under the curves (AUCs) for receiver operating curves (ROCs) were compared. The feature importance maps from XGBoost and lightGBM were sparse and their performance were comparable, so we opted to utilize ridge logistic regression for the remainder of the study.

In Methodology 1, voxel doses were the only input features for the models, with the pharyngeal constrictors and larynx analyzed in separate models. The data set was randomly split into training and test sets in an 80-20% ratio, and the same training patients were used for both pharyngeal constrictors and larynx models. The hyperparameter was tuned using the Optuna package (73) over the 5-fold cross-validation, with ROC AUC as the performance metric. Accuracy and F1 scores evaluated at the threshold of 0.5 were also utilized to compare the general performance of the models. Full training data was used to perform the final fit with the optimized hyperparameter to create the model. For the 3D models, the standardized input features allowed the coefficients from the ridge logistic regression to directly signify feature importance. Specifically, each coefficient from the regression showcases the change in log odds of the outcome for a unit increase in its respective feature. These feature importance coefficients were qualitatively reviewed in a scatter plot for any sub-regions that differentiated FT/WL better than others. In addition to ridge logistic regression, other regularized methods such as LASSO and elastic net were also attempted. However, these L1-based regularization, promoting sparsity combined with highly correlated input parameters yielded spatially fragmented and variable feature importance maps. Therefore, ridge regression was preferred to retain spatial coherence for subregion identification.

**Figure 3:**
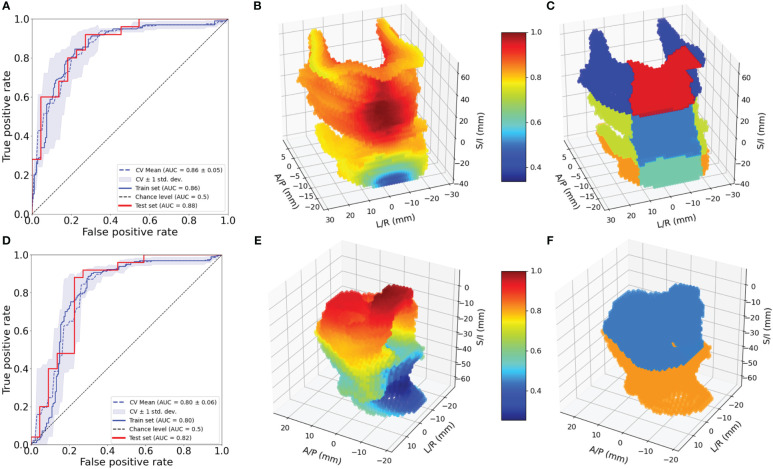
Evaluation of 3D models for pharyngeal constrictor and larynx. ROC for pharyngeal constrictor **(A)** and larynx **(D)** 3D models. Feature importance patterns for pharyngeal constrictor **(B)** and larynx **(E)**. Subregions of pharyngeal constrictor **(C)** and larynx **(F)** used for the aggregate models.

In methodology 2, a univariate logistic regression was first performed for each clinical and demographic variable fitting to the binary outcomes of FT/WL, and p-value and odds ratio were calculated. We first developed a model exclusively incorporating the non-dosimetric clinical variables listed in **Table 2**, which served to establish a baseline for prediction performance. Variables identified as predictive of FT/WL in the univariate analysis were used for modeling with the dosimetric variables. In the final models, there were seven to eight dosimetric variables and six clinical and demographic variables. The clinical and demographic variables were common between the DVH model and the regional dose model: area deprivation index, baseline weight, treatment setting (primary/post-operative), concurrent chemotherapy (yes/no), bilateral treatment (yes/no), and baseline dysphagia grade 0 (yes/no). The dosimetric variables for the DVH metrics model were mean larynx dose, mean pharyngeal constrictor dose, larynx V50Gy and V60Gy, and pharyngeal constrictor V50Gy, V55Gy, and V60Gy, where V*x*Gy represents the percent volume of the organ covered by *x* Gy or more. These DVH metrics were chosen because they are used to evaluate head and neck treatment plans in our clinic. For the regional dose model, the pharyngeal constrictors were divided into superior, middle and inferior pharyngeal constrictors on the reference patient as well as midline and lateral regions as shown in **Figure 3C**, grouping the regions based on the feature importance from the voxel models. Following a similar grouping, the larynx was divided into supraglottic larynx and inferior regions as shown in **Figure 3F**. Mean doses from these subregions were used as the dosimetric variables for the regional dose model. Using these input features, models were trained 50 times with the 5-fold cross-validation process with varying random data splits to confirm that the learned hyperparameters and feature importances were not heavily dependent on a specific random partitioning of the dataset. To assess feature importance, we employed a permutation test (74). We randomized the data of one input variable at a time and evaluated the resulting drop in model performance. The magnitude of performance decline, as measured by the change in ROC AUC from the unaltered data, indicates the importance of that variable to the model’s predictive capability. We conducted this permutation process 50 times for every variable, incorporating 5-fold cross-validation on the training dataset in each iteration. The average change in the AUC was used to assess the difference in performance, and the two-tailed Wilcoxon rank sum test was used to evaluate the statistical significance of this change. To compare the general performance of the regional dose model and the DVH metrics model, test set AUCs from the 50 trials were also compared using the two-tailed Wilcoxon rank sum test.

**Table 2:** P-values and odds ratios from the univariate analysis of non-dosimetric variables correlating with FT/WL.

Non-dosimetric variables	P-value	Odds Ratios [95% CI]
**Treatment setting (primary vs post-operative treatment)**	**<0.0001**	**4.62 [2.55 – 8.38]**
**Bilateral treatment**	**<0.0001**	**6.72 [3.07 – 14.73]**
**Concurrent chemotherapy**	**0.001**	**2.54 [1.44 – 4.49]**
**Baseline weight**	**0.01**	**1.014 [1.000-1.028]**
**Area deprivation index**	**0.02**	**1.014 [1.002-1.026]**
**Baseline dysphagia grade = 0**	**0.03**	**0.51 [0.28 – 0.92]**
Gender	0.16	1.63 [0.83-3.20]
Treatment modality (photon vs proton)	0.17	0.69 [0.41 – 1.17]
Never smoker	0.34	0.78 [0.46 – 1.30]
Age	0.39	0.99 [0.97-1.01]

P-values and odds ratios from the univariate analysis of nondosimetric variables correlating with FT/WL. Variables with p-values below 0.05 (bold) showed a statistically significant correlation with FT/WL placement.

## 3 Results

**Figure 4:**
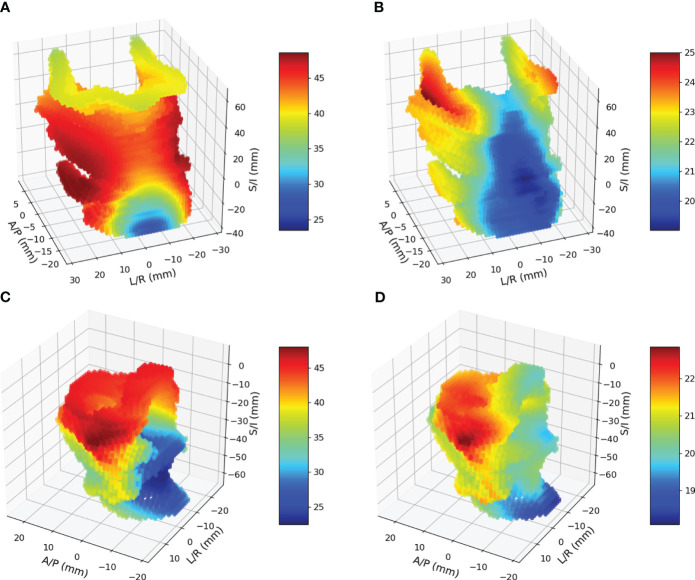
**(A)** and **(B)** are mean and standard deviation of pharyngeal constrictor doses. **(C)** and **(D)** are mean and standard deviations of larynx doses.

Of the 231 patients, 64 patients were found to have utilized FT, and 106 patients lost more than 10% of their baseline weight within 90 days after completing RT. Combining these observations, 122 patients had either FT, WL or both. All registrations in this study yielded a Dice coefficient of greater than 0.7, with a mean and standard deviation of 0.80 ± 0.02 for pharyngeal constrictors and 0.84 ± 0.06 for larynx. **Figure 4A, B** shows the mean and standard deviation of pharyngeal constrictor dose distributions for the entire cohort. The average mean pharyngeal constrictor dose was 52.3 ± 11.3 Gy and 31.7 ± 15.2 Gy for those who experienced FT/WL and those who did not, respectively. **Figure 4C, D** show the mean and the standard deviation of larynx dose distributions. Among those who experienced FT/WL, the average mean larynx dose was 41.6 ± 14.4 Gy while that of patients who did not experience FT/WL was 26.5 ± 18.3 Gy.

### 3.1 Voxel-based dose model

**Figure 3A** shows the ROC curves for the 3D pharyngeal constrictor model. The AUC for the models’ training and test data were found to be 0.87 and 0.86, respectively, with cross-validation yielding 0.86 ± 0.02. The accuracy and F1 scores for the test data were 0.83 and 0.85, respectively. **Figure 3B** shows the feature importance from the model, highlighting the middle to superior pharyngeal constrictors as being more impactful when compared to the inferior region. The midline voxels of the superior and middle pharyngeal constrictors were most important for determining FT/WL for this cohort. **Figure 3D, E** illustrates ROC and the feature importance pattern from the larynx 3D model. The AUC for the model’s training and test data was 0.80 and 0.82, respectively, with cross-validation yielding 0.80 ± 0.06. The accuracy and F1 scores for the test data were both 0.72. The feature importance consistently highlights the superior part of the larynx as a critical factor for predicting FT/WL.

### 3.2 Univariate analysis and aggregated dosimetric model

**Figure 5:**
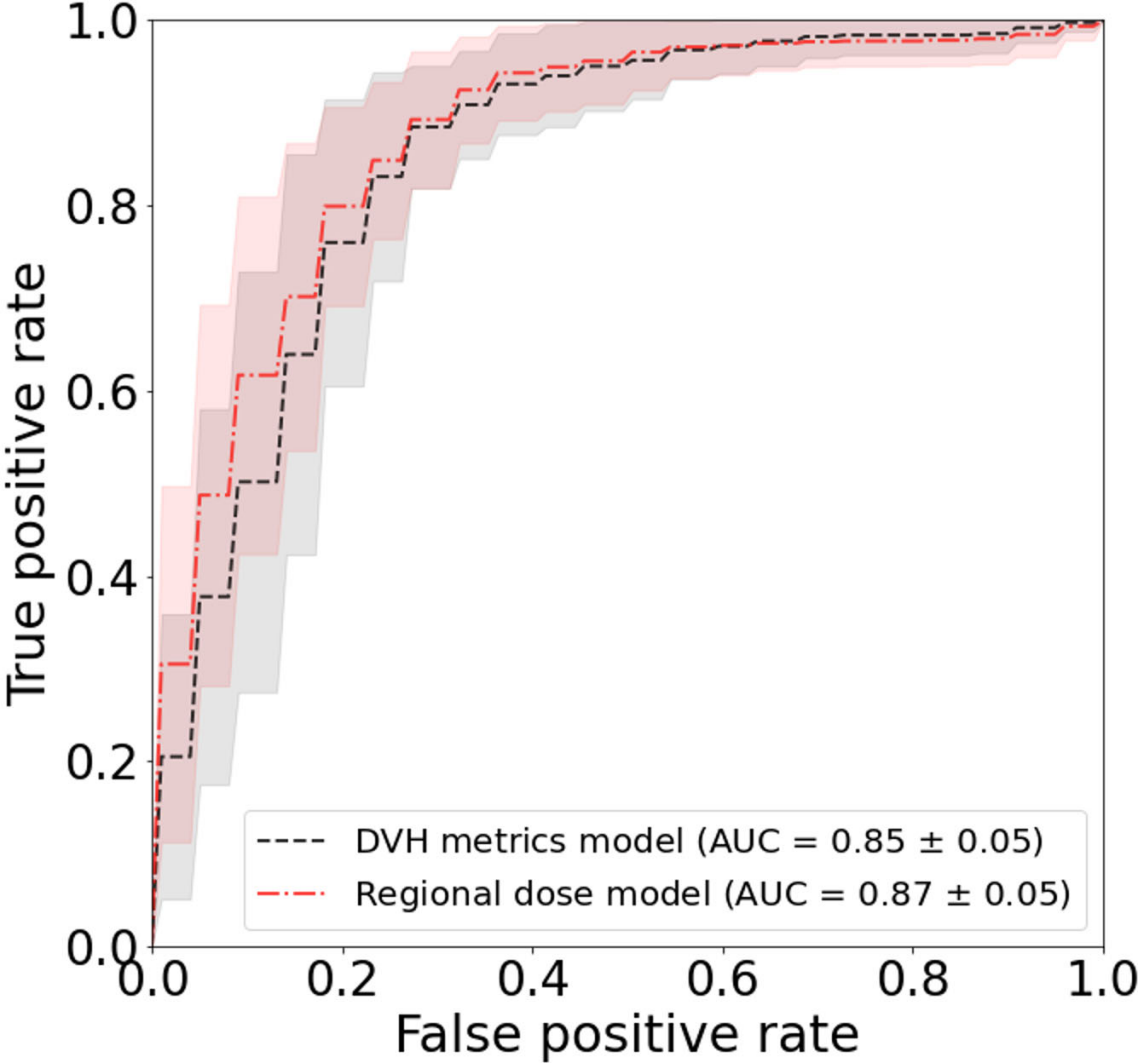
Test set ROC for FT/WL prediction using regional dose and dose-volume histogram (DVH) metrics models. Curves show the average area under the ROC curve (AUC) over 50 trials, with shading indicating standard deviation. The regional dose model (mean AUC 0.87 ± 0.05) outperformed the DVH metrics model (0.85 ± 0.05) with p=0.04.

The results of the univariate fits to non-dosimetric variables are shown in **Table 2**. There were six variables that showed significant correlation with FT/WL: treatment setting (primary/post-operative), treatment laterality (bilateral/unilateral), concurrent chemotherapy (yes/no), baseline dysphagia grade =0, baseline weight, and ADI. These six non-dosimetric variables were used as part of the aggregated dosimetric models. Gender, treatment modality (photon vs proton), smoking status and age did not show significant correlation with FT/WL. A clinical variables-only model using the non-dosimetric variables in **Table 2** achieved an AUC of 0.76 ± 0.01 and 0.74 ± 0.06 for training and test set, respectively. The pharyngeal constrictor and larynx subregions are illustrated in **Figure 3C, F**, respectively. Test set ROCs from the regional dose and DVH metrics models are shown in **Figure 5**. The lines indicate the average of the 50 trials, and the shaded area indicates the standard deviations. The regional dose model, with a mean AUC of 0.87 ± 0.05, demonstrated marginally superior performance compared to the DVH metrics model, which had a mean AUC of 0.85 ± 0.05 with a p-value of 0.04. The F1 scores for both models were 0.82 ± 0.04. The incorporation of dosimetric features in the DVH and regional dose models significantly improved performance over the clinical variables-only model (p < 0.0001). **Table 3** shows the results of a permutation test to evaluate feature importance. Input features that significantly affected the performance of the model when shuffled–as assessed by AUC compared to the actual data–are listed in order of significance. For the regional dose model, the most important features were pharyngeal constrictor doses, particularly the superior to middle region. While dose to the superior part of the larynx significantly contributed to the performance of the regional dose model, none of the larynx DVH metrics showed significant performance gain in the DVH metrics model.

**Table 3:** Evaluation of feature importance based on a permutation test for the DVH metrics and regional dose models. The table lists input features in descending order of significance, based on their impact on model performance assessed by ROC AUC. (PC: Pharyngeal constrictor muscles).

	Features from DVH metrics model	Mean AUC loss	Features from regional dose model	Mean AUC loss
1	PC: Mean dose	-0.021	PC: superior lateral mean dose	-0.022
2	PC: V50Gy (%)	-0.016	PC: superior midline mean dose	-0.020
3	PC: V55Gy (%)	-0.012	PC: middle midline mean dose	-0.011
4	Baseline weight	-0.006	PC: middle lateral mean dose	-0.009
5	PC: V65Gy (%)	-0.006	Baseline weight	-0.004
6	Area Deprivation Index	-0.004	Larynx: superior mean dose	-0.004
7	Concurrent chemotherapy (yes/no)	-0.002	Area Deprivation Index	-0.003
8	Treatment setting	-0.001	Concurrent chemotherapy (yes/no)	-0.002
9	Baseline dysphagia grade = 0 (yes/no)	-0.0006	Baseline dysphagia grade = 0 (yes/no)	-0.0004

## 4 Discussion

This study delves into the relationship between radiation dose distributions to two organs at risk (OARs) and feeding tube use or weight loss in head and neck cancer patients receiving radiotherapy. Employing deformable image registration and ridge logistic regression, we mapped structures and identified key regions, particularly the superior part of the pharyngeal constrictors and the superior part of the larynx, as crucial determinants for FT/WL. This finding was revealed with 3D voxel-based models and validated using aggregated dose model with reduced input features to minimize risk of overfitting.

Our study highlights that dose to the superior pharyngeal constrictor muscles was most important in predicting FT/WL in our cohort. While we did not find studies investigating the same endpoint, our findings are in general agreement with prior clinical (75) and outcomes studies (43) demonstrating the radiosensitivity of sub-regions in organs and their role in post-radiation dysphagia and aspiration. Feng et al. (76) and Eisbruch et al. (38) also reported that highest correlations of videofluoroscopy based aspiration and dysphagia to the superior pharyngeal constrictor in a prospective study. Petras et al. (77) have evaluated the relationship between dose to larynx subregions and swallowing toxicities assessed by aspiration at one year, and Hedstrom et al. (78) considered dysphagia at 6-months post-treatment. Both studies identified the epiglottis as a critical subregion, in line with our findings.

Overall, the performance of the DVH metrics and the regional dose models were similar. This supports our current standard of care of using DVH for treatment planning. However, we note that feature importance analysis for the DVH metrics model revealed that the model performance did not significantly depend on larynx DVH metrics. On the other hand, the aggregated regional dose model utilized mean doses from superior-middle pharyngeal constrictor and supraglottic larynx. This study motivates further investigation into dose sparing of these subregions. Understanding the dose distribution effects will facilitate voxel-based optimization, evaluation and interpretation of treatment plans that have similar DVH metrics.

The strength of our work includes the number of cohorts, standardized contouring of the pharyngeal constrictors and the larynx, as well as inclusion of patients treated with proton therapy. In contrast to the pharyngeal constrictor and larynx segmentation created during clinical workflow, contours used in this study were retrospectively drawn to achieve high consistency and conformance to the contouring guideline. Consequently, the definition of the organ was consistent across the entire patient cohort. For future studies, automatic segmentation using artificial intelligence-driven algorithms along with quality assurance processes could aid in generating more consistent anatomical segmentations than those available from clinical data. However, automatic segmentation algorithms are typically not trained to segment substructures. As this study demonstrated, a voxel-based approach allows us to eliminate the need for exhaustive contouring of each substructure *a priori* for the entire cohort, thereby streamlining the analytical process.

Limitations of our work include the nature of single-institution studies and the lack of a treatment planning component to the study, so that it remains to be seen what amount of dose reduction to the mid to superior pharyngeal constrictor and supraglottic larynx could be achieved without compromising treatment quality. An additional limitation is the lack of controlled study on treatment modality (proton/photon). The relative biological effectiveness (RBE) of 1.1 (58–60) for protons used in this study applies to tumor control outcomes, which may not directly translate to functional outcomes in normal tissues. Furthermore, our study included 21 photon patients who received lower prescription doses of 30 Gy. Since our primary objective was to investigate effects of various dose distributions, we opted to include those treatments to increase diversity. However, these choices likely confounded the analysis with respect to treatment modality. While promising, our findings warrant validation in diverse cohorts, treatment modality, treatment planning techniques, and treatment regimens. Nevertheless, our study highlights the potential of interpretable voxel-based modeling to elucidate impact of inhomogeneous dose distributions within an organ.

## 5 Conclusion

In conclusion, the 3D voxel-based analysis and the aggregated regional dose analysis highlighted the superior subregion of the pharyngeal constrictor muscles and the supraglottic larynx as the most important predictor of FT/WL within 90 days of RT.

## Data availability statement

The data analyzed in this study is subject to the following licenses/restrictions: protected health information. Requests to access these datasets should be directed to shiraishi.satomi@mayo.edu.

## Ethics statement

The studies involving humans were approved by Mayo Clinic Institutional Review Board. The studies were conducted in accordance with the local legislation and institutional requirements. Written informed consent for participation in this study was provided by the participants’ legal guardians/next of kin.

## Author contributions

SM: Data curation, Investigation, Methodology, Visualization, Writing – original draft. MG: Writing – review & editing. YG: Writing – review & editing. SL: Writing – review & editing. DJM: Writing – review & editing. DWM: Writing – review & editing. MW: Writing – review & editing. JQ: Methodology, Writing – review & editing. DR: Writing – review & editing. RF: Conceptualization, Data curation, Writing – review & editing. SS: Conceptualization, Data curation, Formal analysis, Funding acquisition, Investigation, Methodology, Software, Supervision, Validation, Visualization, Writing – original draft.
